# Development of risk prediction models to predict urine culture growth for adults with suspected urinary tract infection in the emergency department: protocol for an electronic health record study from a single UK university hospital

**DOI:** 10.1186/s41512-020-00083-2

**Published:** 2020-09-16

**Authors:** Patrick Rockenschaub, Martin J. Gill, David McNulty, Orlagh Carroll, Nick Freemantle, Laura Shallcross

**Affiliations:** 1 Institute of Health Informatics, University College London, 222 Euston Road, London, NW1 2DA UK; 2 Department of Microbiology, Queen Elizabeth Hospital Birmingham, University Hospitals Birmingham NHS Foundation Trust, Mindelsohn Way, Birmingham, B15 2TH UK; 3 Health Informatics, University Hospitals Birmingham NHS Foundation Trust, 11-13 Frederick Road, Edgbaston, Birmingham, B15 1JD UK; 4 Department of Medical Statistics, London School of Hygiene and Tropical Medicine, Keppel Street, London, WC1E 7HT UK; 5 Institute of Clinical Trials and Methodology, University College London, 90 High Holborn, London, WC1V 6LJ UK

**Keywords:** Protocol, Diagnosis, Urinary tract infection, Prediction models, Hospital

## Abstract

**Background:**

Urinary tract infection (UTI) is a leading cause of hospital admissions and is diagnosed based on urinary symptoms and microbiological cultures. Due to lags in the availability of culture results of up to 72 h, and the limitations of routine diagnostics, many patients with suspected UTI are started on antibiotic treatment unnecessarily. Predictive models based on routinely collected clinical information may help clinicians to rule out a diagnosis of bacterial UTI in low-risk patients shortly after hospital admission, providing additional evidence to guide antibiotic treatment decisions.

**Methods:**

Using electronic hospital records from Queen Elizabeth Hospital Birmingham (QEHB) collected between 2011 and 2017, we aim to develop a series of models that estimate the probability of bacterial UTI at presentation in the emergency department (ED) among individuals with suspected UTI syndromes. Predictions will be made during ED attendance and at different time points after hospital admission to assess whether predictive performance may be improved over time as more information becomes available about patient status. All models will be externally validated for expected future performance using QEHB data from 2018/2019.

**Discussion:**

Risk prediction models using electronic health records offer a new approach to improve antibiotic prescribing decisions, integrating clinical and demographic data with test results to stratify patients according to their probability of bacterial infection. Used in conjunction with expert opinion, they may help clinicians to identify patients that benefit the most from early antibiotic cessation.

## Background

Urinary tract infection (UTI) is a leading cause of hospital admissions, accounting for 16% of all avoidable emergency admissions [1]. UTI presents with a clinical spectrum that ranges from urosepsis and pyelonephritis to mild urinary symptoms, each of which merits different durations of antibiotic treatment or potentially no antibiotics at all [2, 3]. The diagnosis of UTI syndromes is based on a combination of symptoms and microbiological culture of urine (bacteriuria) and/or blood (bacteraemia) [4]. Obtaining microbiological results introduces a bottleneck for evidence-based diagnosis, since cultures often take 48–72 h to grow. In the meantime, patients are often treated with antibiotics. Previous studies have found that up to 50% of such antibiotic use is unnecessary [5–7]. A wide range of additional information is collected as part of routine hospital care, which may provide an opportunity to reduce the diagnostic uncertainty introduced by the delay in culture results. Stored within electronic health records (EHR), these auxiliary data may help to create risk prediction models that can be used to predict the likely culture result and identify patients who are highly unlikely to have bacterial UTI.

We are aware of very few studies that have looked into using routine health data to predict bacteriuria in emergency department (ED) settings [8, 9]. In a recent study, Taylor et al. predicted bacterial growth in urine sampled from more than 80,000 patients with potential UTI symptoms in four US EDs [8]. Their best performing model achieved an area under the receiver operating characteristic curve (AUROC) of 0.90, with a sensitivity of 61.7% and a specificity of 94.9%. However, there are several reasons why it is difficult to apply this model in an NHS hospital including inclusion of urinalysis results that are not regularly performed in the UK, a relatively broad definition of the population at risk and the exclusion of microbiological culture of blood. In the only other study that we are aware of that attempted to predict bacteriuria in the ED, Wigton et al. achieved a lower AUROC of 0.78 on a sample of 506 patients [9]. Several further studies were performed in primary care settings [10–14] but their generalisability to a generally sicker ED population is questionable.

In this study, we will expand on previously published work [8, 9] and develop a model which aims to judge the probability of bacterial UTI in UK patients who present with suspected UTI in the ED. The models will be developed and tested using data on individuals presenting in the ED at Queen Elizabeth Hospital Birmingham (QEHB). QEHB has EHR which are ideally suited for this purpose, containing high-quality and detailed information on diagnoses, outcomes, investigations, vital signs, drug treatments and diagnostic coding dating back to 2011 [15]. Using these hospital records, our model aims to predict the probability that urinary pathogens will grow in urine and/or blood cultures collected during ED attendance. For admitted patients, additional predictions will be made at specific intervals throughout the first three days of their hospital stay to investigate whether additional information gathered during their inpatient stay, but before availability of culture results, allows to predict culture growth with increased certainty. Finally, we will explore differences in model performance and clinical progression for important subpopulations including the elderly and patients with a recorded alternative infective syndrome (e.g. pneumonia) at arrival or discharge, which do not require antibiotics for UTI but may need them for the treatment of the other infection.

## Aims and objectives

### Aim

To use EHR data from a large UK teaching hospital to predict patients’ probability of bacterial UTI at arrival among individuals with suspected UTI in the ED.

### Objectives

- To develop models that predict bacterial growth in urine and/or blood samples collected during ED attendance based on clinical information recorded in the patient’s medical history and in the ED
- To assess the change in predictive performance at pre-defined times after admission (0, 12, 24, 36, 48, 60 and 72 h) to determine whether additional inpatient data collected up to 72 h after admission to hospital leads to increased predictive certainty
- To compare the predictive performance of each model in different subpopulations, considering sex, age, clinical syndrome (lower UTI, pyelonephritis, urosepsis), final diagnoses (UTI, other infection, non-infective diagnosis) and risk of complications (death, admission to intensive care, length of stay)
- To evaluate the expected performance of our models in temporally and/or geographically independent patient populations

## Methods/design

### Source of data

QEHB is part of University Hospitals Birmingham NHS Foundation Trust, one of the largest teaching hospitals in England. The trust serves a population of more than 2.2 million patients per year, a large proportion of whom are seen at QEHB [16]. Detailed information on all patients admitted to QEHB is recorded within its electronic patient management system, including clinical diagnoses, observations, assessments and laboratory results [15]. Unlike many other trusts in England, QEHB has also recorded drug prescriptions electronically for more than 10 years, making it an invaluable resource for research linked to antibiotic prescribing.

#### Development dataset

To develop the predictive models, we will use data from all eligible patients who attended the ED at QEHB between 1 November 2011 and 31 December 2017 (electronic recording of ED diagnosis at QEHB started after a system change at the end of October 2011).

#### Validation dataset

We will use data collected at QEHB between 1 January 2018 and 31 March 2019 to externally validate the model. Patients who were included in the development dataset due to an earlier attendance will be excluded from the validation dataset. We will undertake external validation of our models in an independent dataset from University College London Hospitals NHS Foundation Trust.

### Participants

#### Inclusion and exclusion criteria

All patients who attended the ED at QEHB within the study period and who had a urine sample submitted for microbiological testing within 24 h of arrival are eligible for inclusion in the study. A window of 24 h was chosen to account for discrepancies between when the sample was collected and when the urine sample was recorded in the laboratory system (particularly overnight). Patients enter the study at registration in the ED and exit the study on the earliest of the following dates: date of discharge, date of death, date of transfer to a different hospital or date of urine culture results.

Individuals aged < 18 years, pregnant women, patients who were not admitted via the ED and patients whose urine sample was submitted for culture but was not cultured due to standard laboratory protocols at QEHB (see the “Outcome” section for details) will be excluded from the analysis.

### Outcome

The principal outcome of interest is microbiological growth (≥ 10^4^ colony-forming units/mL). Only urine samples that were eventually cultured will be included in the analysis. Microbiological cultures at QEHB are performed in accordance with standard laboratory procedures (UK Standards for Microbiology Investigations: SMI B41, Investigation of Urine; SMI B37, investigation of blood cultures (for organisms other than *Mycobacterium* species) [17]. The decision whether to culture a urine sample depends on cell count results performed in the laboratory. Only urines with white blood cell counts and bacteria counts above a threshold value were cultured. At the start of the study, the threshold value for proceeding to culture was white cell counts > 40/μL or bacteria counts > 4000/μL. This was adjusted to white cell counts > 80/μL or bacteria counts > 8000/μL following the introduction of a revised standard operating procedure in the microbiology laboratory in October 2015. Performing cell counts is not possible for urine samples less than 4 mL or for samples too viscous to pass through the instrument. Samples for which cell counts could not be performed are always cultured and included in the analysis. Following the standard procedure at QEHB, (heavy) mixed growth in the urine sample will be considered as contamination, except where *E. Coli* was present. In addition, samples will be classified as positive if there are < 10^4^ colony-forming units/mL but the same urinary pathogen is identified from a blood culture, implying urosepsis.

### Predictors

We will consider a wide range of candidate predictors relating to characteristics of the urine sample, a patient’s clinical presentation at the start of and throughout the hospital stay, and to risk factors encoded in a patient’s medical history (Table 1). Candidate predictors were chosen based on clinical experience, the frequency with which variables are measured in the clinical context where the model is likely to be applied, and existing literature [8].

**Table 1:** Candidate predictors of bacteriuria measured (a) once at admission (constant throughout one hospital stay; time independent) and (b) multiple times throughout a patient’s hospital stay (time-dependent)

Candidate predictor	Definition	Units/categories
(a) Measured at admission		
Demographic		
Age	Recorded age at hospital admission in 10-year age bands (continuous age is unavailable due to privacy regulations)	18–24, 25–34,…, 95–104
Sex	As recorded in the admission notes	Male/female
Social deprivation	Index of Multiple Deprivation (IMD) 2015 quintile	Deciles (1–10)
Ethnicity	As recorded in the admission notes; collapsed into 5 major categories	Asian, Black, Mixed, other, White
Co-morbidity		
Charlson Co-morbidity Index	Numeric comorbidity score based on the presence of relevant ICD-10 codes in the entire hospital record*	Count (1–33)
Underlying renal disease	Presence of a relevant ICD-10 code in the previous 5 years*	Yes/no
Underlying urological condition	Presence of a relevant ICD-10 code in the previous 5 years*	Yes/no
Renal or urological surgery	Presence of a relevant OPCS code in the previous 5 years*	Yes/no
Immunosuppression	Presence of a relevant ICD-10 code in the prior year*	Yes/no
Cancer	Presence of a relevant ICD-10 code in the prior year*	Yes/no
Previous healthcare contact		
Discharge from hospital in prior 7 days	Most recent discharge date from QEHB within 7 days of index attendance date	Yes/no
Number of previous admissions	Number of hospital spells at QEHB in the prior year	Count (≥ 0)
Number of days spent in hospital	Number of days spent as an inpatient at QEHB in the prior year	Count (≥ 0)
Number of previous ED attendances	Number of ED attendances at QEHB in the prior year	Count (≥ 0)
Factors predisposing to UTI		
Previous admission for UTI	Admission to QEHB with an ICD-10 code of UTI on discharge in the prior year*	Yes/no
Previous ED attendance for UTI	ED attendance at QEHB with ED diagnosis of lower UTI, pyelonephritis or urosepsis in the prior year	Yes/no
Number of previous admissions for UTI	Number of hospital spells at QEHB with an ICD-10 code of UTI on discharge in the prior 2 years*	Count (≥ 0)
Number of previous ED attendances for UTI	Number of ED attendances at QEHB with ED diagnosis of lower UTI, pyelonephritis or urosepsis in the prior year*	Count (≥ 0)
Previous urine culture	Urine sample submitted at QEHB for microbiological diagnosis in prior year	Yes/no
Previous bacteriuria	Urinary pathogen identified at QEHB from blood or urine in prior year	Yes/no
Previous resistant pathogen	Drug-resistant pathogen identified at QEHB from blood or urine in prior year	Yes/no
Prior antibiotic consumption	Total antibiotic consumption in QEHB in prior year	Defined daily doses (DDDs) (≥ 0) [18]
Characteristics of the admission		
Admitted from care home	As recorded	Yes/no
Month of admission	As recorded	January,…, December
Day of year of admission	As recorded	Count (1–366)
Day of week of admission	As recorded	Monday,…, Sunday
Investigations in the ED		
Suspected diagnosis in the ED	ED impression of clinical syndrome as recorded by the ED clinician	Lower UTI, pyelonephritis, urosepsis
Positive urinalysis	Presence of leucocytes and/or nitrates in urinalysis	Yes/no
Urinalysis		
Leucocytes	As recorded by the clinician (dipstick test)	Positive/negative
Nitrates	As recorded by the clinician (dipstick test)	Positive/negative
White blood cells	As recorded by the laboratory (flow cytometry)	Count/μL
Red blood cells	As recorded by the laboratory (flow cytometry)	Count/μL
Epithelial cells	As recorded by the laboratory (flow cytometry)	Count/μL
Small round cells	As recorded by the laboratory (flow cytometry)	Count/μL
Bacteria	As recorded by the laboratory (flow cytometry)	Count/μL
Yeast	As recorded by the laboratory (flow cytometry)	Count/μL
Conductivity	As recorded by the laboratory (flow cytometry)	mS/cm
Casts	As recorded by the laboratory (flow cytometry)	Count/μL
Crystals	As recorded by the laboratory (flow cytometry)	Count/μL
(b) Measured multiple times throughout hospital stay^†^		
Clinical observations		
Heart rate	As recorded	Beats per minute
Respiratory rate	As recorded	Breaths per minute
Body temperature	As recorded	C°
Oxygen saturation	As recorded	Percent
Systolic blood pressure	As recorded	mmHg
AVPU	As recorded	Alert, verbal, pain, unresponsive
SEWS	Standardised Early Warning Score as recorded or calculated based on heart rate, respiratory rate, body temperature, oxygen saturation and AVPU	Count (0–18)
Clinical investigations		
White cell count (blood)	As recorded	10^3^/mL
C-reactive protein	As recorded	mg/L
Creatinine	As recorded	μmol/L
Acute kidney injury score	Defined as the change in serum creatinine compared to an approximate baseline measure (i.e. average creatinine in previous 6 months)	Stage 0 (1.0–1.5 × baseline), stage 1 (1.5–1.9), stage 2 (2.0–2.9) stage 3 (≥ 3.0)
Alkaline phosphatase	As recorded	IU/L
Bilirubin	As recorded	μmol/L
Platelets	As recorded	10^9^/L
Antibiotic treatment		
Antibiotic treatment	Recorded administration of any systemic antibiotic (British National Formulary chapter 5.1.^**‡**^)	Yes/no
Broad-spectrum antibiotic	Recorded administration of any of the following antibiotics: co-amoxiclav, piperacillin-tazobactam, carbapenems, cephalosporins (except 1st generation), quinolones, colistin, fosfomycin, aminoglycosides	Broad-spectrum, narrow-spectrum, none
Route of administration	Recorded route of administration, giving precedence to intravenous (IV) antibiotics (i.e. if multiple antibiotics are prescribed with ≥ 1 IV, treatment is classified as IV)	IV, oral, none
Dosage	As recorded	DDDs (≥ 0) [18]

*Detailed code lists available in the appendix

^†^For each time-dependent variable, we will also consider the change in value compared to the last observed measurement

^‡^Excluding anti-tuberculosis and anti-leprosy medication

### Sample size

Each year, around 60,000 patients are seen in the ED at QEHB. In 2014, more than 4500 patients were admitted to QEHB and prescribed an antibiotic. Preliminary analysis suggests that 20% of these prescriptions were for suspected UTI syndromes; hence, we expect ~ 5400 admitted patients using data from late 2011 to end of 2017 (6 years) [19]. Based on clinical experience, we expect a similar number of patients with suspected UTI syndromes to be discharged directly from the ED, resulting in an estimated total training sample of ~ 10,800 patients. Assuming a prevalence of bacteriuria of 30% like that reported by Taylor et al. previously, this would imply > 30 events per variable when including all variables defined in Table 1.

### Statistical analysis methods

#### Feature engineering and selection

All continuous predictors will be winsorized at the 1st and 99th percentile to account for outliers and normalised to lie within the range (0, 1). Categorical predictors will be encoded in a full-rank encoding, combining levels with a small number of cases (< 5%). Predictors with zero variance will be excluded before analysis. For highly correlated predictors (correlation coefficient > 0.9 using Spearman’s rank correlation), one predictor will be removed before analysis based on clinical judgement. Similarly, predictors which are found to be largely missing and might thus not be expected to be present when the model will be used in practice at QEHB will be removed from the analysis before fitting the models.

We will consider the use of fractional polynomials (FP) with up to four degrees of freedom (i.e. 2 fractional polynomial terms) for each numerical predictor [20, 21]. We will estimate the optimal number of FPs using the Akaike Information Criterion. Once the best-fitting FPs have been determined, we will consider models with all predictors and parsimonious models selected via backwards feature elimination based on Wald statistics and Rubin’s rules [22]. Since the large number of possible predictors might limit the model’s usability in clinical practice, we follow Taylor et al. and consider a minimal model based on age, sex, urinalysis results and history of UTI [8].

#### Type of model

##### Baseline model in the ED

We will first develop a multivariable logistic regression model to predict bacterial growth in the urine and/or blood sample at the end of ED attendance. A prediction will be made for each patient based on the fitted value, which will serve as a baseline comparison for all further models considered.

##### Landmarking models at distinct time points after hospital admission

Additional measurements taken during the first couple of days in hospital may further improve the predictive power of our risk prediction models. We will develop a set of landmarking logistic regression models [23] that predict the probability of bacterial growth in the ED urine sample at pre-defined times *t* = {0, 12, 24, 36, 48, 60} hours after the patient has left the ED and was admitted to the hospital ward. In order to do so, we require a value for each included predictor at time *t*. Since predictors are measured irregularly throughout the patient’s hospital stay, we will first train a multivariate generalized linear mixed model (MGLMM) on all past predictor values up to time *t* to estimate the most likely value of each predictor at time *t* (see the “Missing data” section below for details). Values at time *t* will be estimated using the best linear unbiased predictors from the empirical Bayes posterior distribution of the random effects, conditional on past predictor measurements [23]. The estimated predictor values will then be fed to a logistic regression model that predicts the probability of microbiological growth in the ED sample after having observed the patient for *t* hours. As a result, patients might have more than one prediction, one for each time *t* at which they were still part of the at-risk population. Only patients still admitted and without a culture result at time *t* will be considered at-risk and will be included in the fitting and evaluation of the logistic regression model for time *t*.

#### Missing data

In EHR data, information is only recorded when events take place and we cannot distinguish between cases in which a test or diagnosis was not made and cases in which they were made but not recorded. Consequently, if historical variables such as co-morbidities, procedures, admission records, test results and procedures are not recorded (e.g. because they were performed at another hospital), we will have to assume that these events did not take place. For other variables with missing values that should have been obtained during the current visit (particularly vital signs and laboratory measurements), we will examine the pattern of missingness and impute values where appropriate depending on the type of prediction model.

Our baseline model is a logistic regression, which requires a non-missing value for each included predictor. We will use multivariate imputation by chained equations (MICE) based on the assumption that data are missing at random, i.e. whether a variable is missing or not only depends on the values of observed variables [24]. Following standard MICE procedures [25], we will include all predictors as well as the prediction outcome in the imputation procedure and impute 5 datasets with 10 iterations per dataset (Table 2). Depending on computational feasibility, we will aim to impute up to 100 datasets for our final model to ensure that we obtain robust imputations. Model training will be performed on the imputed development dataset. However, we cannot use the same imputation procedure to evaluate our models since we expect predictors to also be missing during model deployment. When used in practice, our model must impute any missing data in real-time before making a prediction, but at this point, no outcome will be available yet to use in the imputation. This will tend to result in suboptimal imputations when the model is used in practice [25]. To obtain an honest estimate of the performance of our models, we will evaluate them on a second set of imputations that were fit without using the outcome in the imputation procedure, emulating the situation in which the model will ultimately be used [26].

**Table 2:** Conditional models used in the multivariate imputation by chained equations

Variable type	Conditional model
Continuous	Predictive mean matching with type 1 matching and 10 donors
Binary	Logistic regression
Multinomial	Polytomous regression

For our time-dependent models, the nature of missing data slightly differs. Values for each predictor might have been recorded never, once or multiple times before time *t* and we are interested in estimating the most likely value at time *t*. To estimate a good approximation for each predictor, we will separately fit a MGLMM at each landmarking time [23]. Each model will include fixed intercepts and slopes for each predictor and a time-dependent covariate indicating concurrent antibiotic treatment. We will consider correlation structures of varying complexity, with uncorrelated and correlated patient-specific random intercepts and/or slopes for each predictor. If the MGLMM is intractable, we will consider a simpler last observation carried forward (LOCF) method to estimate predictor values at time *t*, or a mixture of LOCF and MGLMM.

#### Model validation

Clinical diagnosis of bacterial UTI requires the presence of urinary symptoms in addition to microbiological culture. Bacteriuria in the absence of urinary symptoms (called asymptomatic bacteriuria) should not be treated with antibiotics [2]. Prevalence of asymptomatic bacteriuria differs between patient groups and increases for example with age. Whereas a urine sample might be sent for culture in many different patients “just in case”, a clinically usable model to confirm or rule out suspected bacterial UTI needs to perform especially well in patients with urinary symptoms. In our main analysis, we will therefore validate our models in the subgroup of patients with a suspected ED diagnosis of lower UTI or pyelonephritis, and our final model will be chosen based on the performance in this group. This group differs from the training population, which will include all patients irrespective of ED diagnosis to increase sample size and provide our model with enough power to learn general relationships. In a secondary analysis, we will also evaluate the performance of our models in patients without an ED diagnosis of UTI as well as in different age groups, by sex and by outcome (i.e. discharge diagnosis, death, admission to intensive care unit, length of stay). We will further consider training our model using only data from patients with a suspected ED diagnosis of lower UTI or pyelonephritis for training to ensure that a heterogeneous training population is not obscuring important relationships in patients with suspected UTI. Finally, we will perform secondary analyses limited to the first visit of each patient and to data after 2015, assessing the impact of repeated patient visits and the impact of increased culture thresholds on our models.

##### Internal validation

Model discrimination in each scenario will be assessed via multiple performance metrics: AUROC, Brier score, area under the precision-recall curve (AUPRC), specificity and negative predictive value (NPV). We will estimate each model’s specificity and NPV at a pre-set sensitivity of 95%, which will evaluate the model’s ability to be used as a screening tool to rule out bacterial UTI. We will assess how well predicted and observed probabilities correspond within each predicted decile (model calibration) by creating a calibration plot and estimating the calibration slope. An estimated slope > 1 indicates underfitting, whereas a slope < 1 indicates overfitting.

Evaluating the model only on the development dataset or a single validation dataset leads to optimistic estimations of the true model performance (henceforth called the apparent performance) [27]. To obtain a more reliable estimate of model performance, we will draw at least 100 bootstrap samples of the development dataset. Where computation time allows for it, we will consider up to 1000 bootstrap samples. All preprocessing and analysis steps including missing data imputation, estimation of fractional polynomials, feature selection and model evaluation will be carried out independently within each bootstrapped sample to avoid any data leakage [28]. The result will be one final model per bootstrapped sample. Evaluating each model on the bootstrap sample in which it was developed provides another estimate of the apparent performance, this time within the bootstrap. To estimate the magnitude of optimism in this bootstrapped apparent performance, we will simultaneously evaluate the bootstrapped model in the original development dataset (called test performance). The difference between test performance and bootstrapped apparent performance will be an estimate of model optimism.

Averaging estimates of the optimism across all bootstrapped samples results in a stable estimate of the optimism [27]. The final, optimism-corrected (“true”) estimate of model performance will then be calculated as follows:

$$ \mathrm{performance}=\mathrm{apparen}{\mathrm{t}}_{\mathrm{original}}-\mathrm{mean}\left(\mathrm{optimism}\right)=\mathrm{apparen}{\mathrm{t}}_{\mathrm{original}}-\frac{1}{B}{\sum}_{b=1}^B\left(\mathrm{apparen}{\mathrm{t}}_b-\mathrm{tes}{\mathrm{t}}_{\mathrm{original}}\right) $$

All metrics used in the model evaluation (AUROC, AUPRC, specificity and NPV) will be adjusted for optimism.

##### External validation

The performance of the model (AUROC, AUPRC, specificity and NPV) in a new dataset will be evaluated using EHRs from patients with suspected UTI who were admitted to QEHB between 1 January 2018 and 31 March 2019. We will summarise average performance and calibration in this temporally independent sample. We will further validate the model in a geographically independent sample of patients from University College London Hospitals NHS Foundation Trust.

All analyses will be performed using the statistical software R [29] including but not necessarily limited to the packages: *tidyverse* [30], *tidymodels* [31], *mice* [32] and *mfp* [33].

## Discussion

The need to reduce inappropriate antibiotic prescribing in secondary care is widely acknowledged, but progress is thwarted by the lack of rapid and reliable diagnostic tests for bacterial infection. Risk prediction models using data contained within EHR offer a new approach to improve antibiotic prescribing decisions, by integrating clinical and demographic data with test results to stratify patients according to their likelihood of bacterial infection.

However, diagnostic uncertainty represents a major obstacle in the application of risk prediction models for bacterial infection. Clinical infection syndromes often overlap, and diagnoses are often not confirmed by microbial culture. This makes it difficult to reliably distinguish infection from non-infectious conditions, but also to discriminate between clinical infection syndromes.

For these reasons, we have not attempted to develop a model which supports decision around antibiotic initiation in the ED, recognising that few doctors will be willing to withhold antibiotics if patients are unwell and the diagnosis is uncertain. Instead, we have opted for a model that identifies patients who may benefit from early antibiotic cessation since they are actually at low risk of bacterial UTI. Descriptive analyses of patients who have been categorised by the model as low/high risk of bacterial UTI will identify categories of patients who are most likely to be low risk, for example based on age, sex and UTI syndrome at presentation. This will be used in conjunction with expert clinical opinion to define a “low-risk” population of patients who have been treated with antibiotics for suspected UTI but are unlikely to benefit from antibiotic treatment. Individuals from this population sub-group will be asked to participate in a proof of concept trial, and randomised to either stop antibiotics early, or to continue antibiotic as per standard care. The trial will assess the safety and feasibility of early antibiotic cessation in these patients and lay the foundation for a future multi-centre trial. It will also demonstrate the potential use of EHR datasets to guide prescribing decisions.

## Supplementary information

**Additional file 1:.** Codelist

## Data Availability

The data that support the findings of this study are available from University Hospitals Birmingham NHS Foundation Trust, but restrictions apply to the availability of these data, which are not publicly available. Data are however available from the authors upon reasonable request and with permission of University Hospitals Birmingham NHS Foundation Trust. Codelists can be found in the appendix and analytical code used in the study will be made available by the authors.

## Abbreviations

AUPRC: Area under the precision-recall curve
AUROC: Area under the receiver operating characteristic curve
CCI: Charlson Co-morbidity Index
DDD: Defined daily dose
ED: Emergency department
EHR: Electronic health records
FP: Fractional polynomials
ICD-10: International Classification of Diseases 10th Revision
IMD: Index of Multiple Deprivation
IV: Intravenous
LOCF: Last observation carried forward
MGLMM: Multivariate generalized linear mixed model
MICE: Multivariate imputation by chained equations
NHS: National Health Service
NPV: Negative predictive value
OPCS: Classification of Interventions and Procedures version 4
QEHB: Queen Elizabeth Hospital Birmingham
SMI: UK Standards for Microbiology Investigations
UTI: Urinary tract infection

## References

[1] Blunt I. Focus on preventable admissions: trends in emergency admissions for ambulatory care sensitive conditions, 2001 to 2013. The Health Foundation and The Nuffield Trust; 2013.

[2] Urinary tract infection (lower): antimicrobial prescribing. NICE guideline [NG109]. National Institute of Health and Care Excellence. https://www.nice.org.uk/guidance/ng109/chapter/Summary-of-the-evidence. Accessed 28 May 2019.

[3] National Institute of Health and Care Excellence. Pyelonephritis - acute. https://cks.nice.org.uk/pyelonephritis-acute. Accessed 10 Jan 2020.

[4] National Institute for Health and Care Excellence. Urinary tract infection (lower) - women. https://cks.nice.org.uk/urinary-tract-infection-lower-women. Accessed 13 Jan 2020.

[5] Woodford HJ, George J (2009). Diagnosis and management of urinary tract infection in hospitalized older people. J Am Geriatr Soc.

[6] Tomas ME, Getman D, Donskey CJ, Hecker MT (2015). Overdiagnosis of Urinary Tract Infection and Underdiagnosis of Sexually Transmitted Infection in Adult Women Presenting to an Emergency Department. J Clin Microbiol.

[7] McIsaac WJ, Hunchak CL (2011). Overestimation error and unnecessary antibiotic prescriptions for acute cystitis in adult women. Med Decis Mak.

[8] Taylor RA, Moore CL, Cheung K-H, Brandt C (2018). Predicting urinary tract infections in the emergency department with machine learning. PLoS One.

[9] Wigton RS, Hoellerich VL, Ornato JP, Leu V, Mazzotta LA, Cheng IH (1985). Use of clinical findings in the diagnosis of urinary tract infection in women. Arch Intern Med.

[10] Little P, Turner S, Rumsby K, Warner G, Moore M, Lowes JA (2006). Developing clinical rules to predict urinary tract infection in primary care settings: sensitivity and specificity of near patient tests (dipsticks) and clinical scores. Br J Gen Pract.

[11] McIsaac WJ, Moineddin R, Ross S (2007). Validation of a decision aid to assist physicians in reducing unnecessary antibiotic drug use for acute cystitis. Arch Intern Med.

[12] Heckerling PS, Canaris GJ, Flach SD, Tape TG, Wigton RS, Gerber BS (2007). Predictors of urinary tract infection based on artificial neural networks and genetic algorithms. Int J Med Inform.

[13] Gadalla AAH, Friberg IM, Kift-Morgan A, Zhang J, Eberl M, Topley N (2019). Identification of clinical and urine biomarkers for uncomplicated urinary tract infection using machine learning algorithms. Sci Rep.

[14] Burton RJ, Albur M, Eberl M, Cuff SM (2019). Using artificial intelligence to reduce diagnostic workload without compromising detection of urinary tract infections. BMC Med Inform Decis Mak.

[15] Freemantle N, Ray D, Falcaro M, McNulty D, Shallcross L, Wood J (2016). BMI upon discharge from hospital and its relationship with survival: an observational study utilising linked patient records. J R Soc Med.

[16] National Health Service. University Hospitals Birmingham NHS Foundation Trust. 2009. https://www.nhs.uk/Services/Trusts/Overview/DefaultView.aspx?id=1470. Accessed 17 Dec 2019.

[17] Public Health England. Standards for microbiology investigations (UK SMI). GOV.UK. 2014. https://www.gov.uk/government/collections/standards-for-microbiology-investigations-smi. Accessed 29 Jul 2019.

[18] World Health Organisation. WHOCC - ATC/DDD Index. https://www.whocc.no/atc_ddd_index/. Accessed 17 Dec 2019.

[19] Shallcross LJ, Freemantle N, Nisar S, Ray D (2016). A cross-sectional study of blood cultures and antibiotic use in patients admitted from the Emergency Department: missed opportunities for antimicrobial stewardship. BMC Infect Dis.

[20] Morris TP, White IR, Carpenter JR, Stanworth SJ, Royston P (2015). Combining fractional polynomial model building with multiple imputation. Stat Med.

[21] Ambler G, Royston P (2001). Fractional polynomial model selection procedures: investigation of type i error rate. J Stat Comput Simul.

[22] Wood AM, White IR, Royston P (2008). How should variable selection be performed with multiply imputed data?. Stat Med.

[23] Paige E, Barrett J, Stevens D, Keogh RH, Sweeting MJ, Nazareth I (2018). Landmark Models for Optimizing the Use of Repeated Measurements of Risk Factors in Electronic Health Records to Predict Future Disease Risk. Am J Epidemiol.

[24] White IR, Royston P, Wood AM (2011). Multiple imputation using chained equations: Issues and guidance for practice. Stat Med.

[25] Moons KGM, Donders RART, Stijnen T, Harrell FE (2006). Using the outcome for imputation of missing predictor values was preferred. J Clin Epidemiol.

[26] Wood AM, Royston P, White IR (2015). The estimation and use of predictions for the assessment of model performance using large samples with multiply imputed data. Biom J.

[27] Steyerberg EW, Harrell FE, Borsboom GJ, Eijkemans MJ, Vergouwe Y, Habbema JD (2001). Internal validation of predictive models: efficiency of some procedures for logistic regression analysis. J Clin Epidemiol.

[28] Smialowski P, Frishman D, Kramer S (2010). Pitfalls of supervised feature selection. Bioinformatics..

[29] R Core Team. R: A language and environment for statistical computing. 2018. https://www.R-project.org/.

[30] Wickham H, Averick M, Bryan J, Chang W, McGowan L, François R (2019). Welcome to the Tidyverse. JOSS..

[31] Kuhn M, Wickham H. tidymodels: Easily Install and Load the “Tidymodels” Packages. 2020. https://CRAN.R-project.org/package=tidymodels.

[32] van Buuren S, Groothuis-Oudshoorn K. mice: Multivariate Imputation by Chained Equations in R. J Stat Softw, Articles. 2011;45:1–67.

[33] Ambler G, Benner A. mfp: Multivariable Fractional Polynomials. 2015. https://CRAN.R-project.org/package=mfp.

