# Supplementary material for: Development of risk prediction models to predict urine culture growth for adults with suspected urinary tract infection in the emergency department: protocol for an electronic health record study from a single UK university hospital
Source: Diagn Progn Res. 2020 Sep 16;4:15. doi: 10.1186/s41512-020-00083-2 (PMC7493920; doi:10.1186/s41512-020-00083-2)
Supplement: Supplementary file 1 — Additional file 1:. Codelist [file 41512_2020_83_MOESM1_ESM.docx]

# Urinary tract infection

| **Lower UTI** | |
| --- | --- |
| N30 | Cystitis |
| N300 | Acute cystitis |
| N308 | Other cystitis |
| N309 | Cystitis, unspecified |
| N390 | Urinary tract infection, site not specified |
| O231 | Infections of bladder in pregnancy |
| O232 | Infections of urethra in pregnancy |
| O233 | Infections of other parts of urinary tract in pregnancy |
| O234 | Unspecified infection of urinary tract in pregnancy |
| O239 | Other and unspecified genitourinary tract infection in pregnancy |
| O862 | Urinary tract infection following delivery |

| **Pyelonephritis** | |
| --- | --- |
| N10 | Acute tubulo-interstitial nephritis |
| N11 | Chronic tubulo-interstitial nephritis |
| N110 | Nonobstructive reflux-associated chronic pyelonephritis |
| N111 | Chronic obstructive pyelonephritis |
| N118 | Other chronic tubulo-interstitial nephritis |
| N119 | Chronic tubulo-interstitial nephritis, unspecified |
| N12 | Tubulo-interstitial nephritis, not specified as acute or chronic |
| N136 | Pyonephrosis |
| N159 | Renal tubulo-interstitial disease, unspecified |
| O230 | Infections of kidney in pregnancy |

# Sepsis

| **Sepsis** | |
| --- | --- |
| A400 | Sepsis due to streptococcus, group A |
| A401 | Sepsis due to streptococcus, group B |
| A402 | Sepsis due to streptococcus, group D |
| A403 | Sepsis due to Streptococcus pneumoniae |
| A408 | Other streptococcal sepsis |
| A409 | Streptococcal sepsis, unspecified |
| A410 | Sepsis due to Staphylococcus aureus |
| A411 | Sepsis due to other specified staphylococcus |
| A412 | Sepsis due to unspecified staphylococcus |
| A413 | Sepsis due to Haemophilus influenzae |
| A414 | Sepsis due to anaerobes |
| A415 | Sepsis due to other Gram-negative organisms |
| A418 | Other specified sepsis |
| A419 | Sepsis, unspecified |
| R572 | Septic shock |
| R650 | Systemic Inflammatory Response Syndrome of infectious origin without organ failure |
| R651 | Systemic Inflammatory Response Syndrome of infectious origin with organ failure |

# Comorbidities: urinary, renal, neoplasm, immunosuppression

| **Underlying renal disease** | |
| --- | --- |
| N00 | Acute nephritic syndrome |
| N01 | Rapidly progressive nephritic syndrome |
| N02 | Recurrent and persistent haematuria |
| N03 | Chronic nephritic syndrome |
| N04 | Nephrotic syndrome |
| N05 | Unspecified nephritic syndrome |
| N06 | Isolated proteinuria with specified morphological lesion |
| N07 | Hereditary nephropathy, not elsewhere classified |
| N08 | Glomerular disorders in diseases classified elsewhere |
| N080 | Glomerular disorders in infectious and parasitic diseases classified elsewhere |
| N081 | Glomerular disorders in neoplastic diseases |
| N082 | Glomerular disorders in blood diseases and disorders involving the immune mechanism |
| N083 | Glomerular disorders in diabetes mellitus |
| N084 | Glomerular disorders in other endocrine, nutritional and metabolic diseases |
| N085 | Glomerular disorders in systemic connective tissue disorders |
| N088 | Glomerular disorders in other diseases classified elsewhere |
| N130 | Hydronephrosis with ureteropelvic junction obstruction |
| N131 | Hydronephrosis with ureteral stricture, not elsewhere classified |
| N132 | Hydronephrosis with renal and ureteral calculous obstruction |
| N133 | Other and unspecified hydronephrosis |
| N134 | Hydroureter |
| N135 | Kinking and stricture of ureter without hydronephrosis |
| N137 | Vesicoureteral-reflux-associated uropathy |
| N138 | Other obstructive and reflux uropathy |
| N139 | Obstructive and reflux uropathy, unspecified |
| N14 | Drug- and heavy-metal-induced tubulo-interstitial and tubular conditions |
| N140 | Analgesic nephropathy |
| N141 | Nephropathy induced by other drugs, medicaments and biological substances |
| N142 | Nephropathy induced by unspecified drug, medicament or biological substance |
| N143 | Nephropathy induced by heavy metals |
| N144 | Toxic nephropathy, not elsewhere classified |
| N15 | Other renal tubulo-interstitial diseases |
| N150 | Balkan nephropathy |
| N151 | Renal and perinephric abscess |
| N158 | Other specified renal tubulo-interstitial diseases |
| N16 | Renal tubulo-interstitial disorders in diseases classified elsewhere |
| N160 | Renal tubulo-interstitial disorders in infectious and parasitic diseases classified elsewhere |
| N161 | Renal tubulo-interstitial disorders in neoplastic diseases |
| N162 | Renal tubulo-interstitial disorders in blood diseases and disorders involving the immune mechanism |
| N163 | Renal tubulo-interstitial disorders in metabolic diseases |
| N164 | Renal tubulo-interstitial disorders in systemic connective tissue disorders |
| N165 | Renal tubulo-interstitial disorders in transplant rejection |
| N168 | Renal tubulo-interstitial disorders in other diseases classified elsewhere |
| N17 | Acute renal failure |
| N170 | Acute renal failure with tubular necrosis |
| N171 | Acute renal failure with acute cortical necrosis |
| N172 | Acute renal failure with medullary necrosis |
| N178 | Other acute renal failure |
| N179 | Acute renal failure, unspecified |
| N18 | Chronic kidney disease |
| N181 | Chronic kidney disease, stage 1 |
| N182 | Chronic kidney disease, stage 2 |
| N183 | Chronic kidney disease, stage 3 |
| N184 | Chronic kidney disease, stage 4 |
| N185 | Chronic kidney disease, stage 5 |
| N189 | Chronic kidney disease, unspecified |
| N19 | Unspecified kidney failure |
| N25 | Disorders resulting from impaired renal tubular function |
| N250 | Renal osteodystrophy |
| N251 | Nephrogenic diabetes insipidus |
| N258 | Other disorders resulting from impaired renal tubular function |
| N259 | Disorder resulting from impaired renal tubular function, unspecified |
| N26 | Unspecified contracted kidney |
| N27 | Small kidney of unknown cause |
| N270 | Small kidney, unilateral |
| N271 | Small kidney, bilateral |
| N279 | Small kidney, unspecified |
| N28 | Other disorders of kidney and ureter, not elsewhere classified |
| N280 | Ischaemia and infarction of kidney |
| N281 | Cyst of kidney |
| N288 | Other specified disorders of kidney and ureter |
| N289 | Disorder of kidney and ureter, unspecified |
| N29 | Other disorders of kidney and ureter in diseases classified elsewhere |
| N290 | Late syphilis of kidney |
| N291 | Other disorders of kidney and ureter in infectious and parasitic diseases classified elsewhere |
| N298 | Other disorders of kidney and ureter in other diseases classified elsewhere |

| **Underlying urological disease** | |
| --- | --- |
| N20 | Calculus of kidney and ureter |
| N200 | Calculus of kidney |
| N201 | Calculus of ureter |
| N202 | Calculus of kidney with calculus of ureter |
| N209 | Urinary calculus, unspecified |
| N21 | Calculus of lower urinary tract |
| N210 | Calculus in bladder |
| N211 | Calculus in urethra |
| N218 | Other lower urinary tract calculus |
| N219 | Calculus of lower urinary tract, unspecified |
| N22 | Calculus of urinary tract in diseases classified elsewhere |
| N220 | Urinary calculus in schistosomiasis [bilharziasis] |
| N228 | Calculus of urinary tract in other diseases classified elsewhere |
| N23 | Unspecified renal colic |
| N301 | Interstitial cystitis (chronic) |
| N302 | Other chronic cystitis |
| N303 | Trigonitis |
| N304 | Irradiation cystitis |
| N31 | Neuromuscular dysfunction of bladder, not elsewhere classified |
| N310 | Uninhibited neuropathic bladder, not elsewhere classified |
| N311 | Reflex neuropathic bladder, not elsewhere classified |
| N312 | Flaccid neuropathic bladder, not elsewhere classified |
| N318 | Other neuromuscular dysfunction of bladder |
| N319 | Neuromuscular dysfunction of bladder, unspecified |
| N32 | Other disorders of bladder |
| N320 | Bladder-neck obstruction |
| N321 | Vesicointestinal fistula |
| N322 | Vesical fistula, not elsewhere classified |
| N323 | Diverticulum of bladder |
| N324 | Rupture of bladder, nontraumatic |
| N328 | Other specified disorders of bladder |
| N329 | Bladder disorder, unspecified |
| N33 | Bladder disorders in diseases classified elsewhere |
| N330 | Tuberculous cystitis |
| N338 | Bladder disorders in other diseases classified elsewhere |
| N34 | Urethritis and urethral syndrome |
| N340 | Urethral abscess |
| N341 | Nonspecific urethritis |
| N342 | Other urethritis |
| N343 | Urethral syndrome, unspecified |
| N35 | Urethral stricture |
| N350 | Post-traumatic urethral stricture |
| N351 | Postinfective urethral stricture, not elsewhere classified |
| N358 | Other urethral stricture |
| N359 | Urethral stricture, unspecified |
| N36 | Other disorders of urethra |
| N360 | Urethral fistula |
| N361 | Urethral diverticulum |
| N362 | Urethral caruncle |
| N363 | Prolapsed urethral mucosa |
| N368 | Other specified disorders of urethra |
| N369 | Urethral disorder, unspecified |
| N37 | Urethral disorders in diseases classified elsewhere |
| N370 | Urethritis in diseases classified elsewhere |
| N378 | Other urethral disorders in diseases classified elsewhere |
| N39 | Other disorders of urinary system |
| N391 | Persistent proteinuria, unspecified |
| N392 | Orthostatic proteinuria, unspecified |
| N393 | Stress incontinence |
| N394 | Other specified urinary incontinence |
| N398 | Other specified disorders of urinary system |
| N399 | Disorder of urinary system, unspecified |
| N40 | Hyperplasia of prostate |
| N41 | Inflammatory diseases of prostate |
| N410 | Acute prostatitis |
| N411 | Chronic prostatitis |
| N412 | Abscess of prostate |
| N413 | Prostatocystitis |
| N418 | Other inflammatory diseases of prostate |
| N419 | Inflammatory disease of prostate, unspecified |
| N42 | Other disorders of prostate |
| N420 | Calculus of prostate |
| N421 | Congestion and haemorrhage of prostate |
| N422 | Atrophy of prostate |
| N423 | Dysplasia of prostate |
| N428 | Other specified disorders of prostate |
| N429 | Disorder of prostate, unspecified |

| **Cancer** | |
| --- | --- |
| C00 | Malignant neoplasm of lip |
| C000 | Malignant neoplasm: External upper lip |
| C001 | Malignant neoplasm: External lower lip |
| C002 | Malignant neoplasm: External lip, unspecified |
| C003 | Malignant neoplasm: Upper lip, inner aspect |
| C004 | Malignant neoplasm: Lower lip, inner aspect |
| C005 | Malignant neoplasm: Lip, unspecified, inner aspect |
| C006 | Malignant neoplasm: Commissure of lip |
| C008 | Malignant neoplasm: Overlapping lesion of lip |
| C009 | Malignant neoplasm: Lip, unspecified |
| C01 | Malignant neoplasm of base of tongue |
| C02 | Malignant neoplasm of other and unspecified parts of tongue |
| C020 | Malignant neoplasm: Dorsal surface of tongue |
| C021 | Malignant neoplasm: Border of tongue |
| C022 | Malignant neoplasm: Ventral surface of tongue |
| C023 | Malignant neoplasm: Anterior two-thirds of tongue, part unspecified |
| C024 | Malignant neoplasm: Lingual tonsil |
| C028 | Malignant neoplasm: Overlapping lesion of tongue |
| C029 | Malignant neoplasm: Tongue, unspecified |
| C03 | Malignant neoplasm of gum |
| C030 | Malignant neoplasm: Upper gum |
| C031 | Malignant neoplasm: Lower gum |
| C039 | Malignant neoplasm: Gum, unspecified |
| C04 | Malignant neoplasm of floor of mouth |
| C040 | Malignant neoplasm: Anterior floor of mouth |
| C041 | Malignant neoplasm: Lateral floor of mouth |
| C048 | Malignant neoplasm: Overlapping lesion of floor of mouth |
| C049 | Malignant neoplasm: Floor of mouth, unspecified |
| C05 | Malignant neoplasm of palate |
| C050 | Malignant neoplasm: Hard palate |
| C051 | Malignant neoplasm: Soft palate |
| C052 | Malignant neoplasm: Uvula |
| C058 | Malignant neoplasm: Overlapping lesion of palate |
| C059 | Malignant neoplasm: Palate, unspecified |
| C06 | Malignant neoplasm of other and unspecified parts of mouth |
| C060 | Malignant neoplasm: Cheek mucosa |
| C061 | Malignant neoplasm: Vestibule of mouth |
| C062 | Malignant neoplasm: Retromolar area |
| C068 | Malignant neoplasm: Overlapping lesion of other and unspecified parts of mouth |
| C069 | Malignant neoplasm: Mouth, unspecified |
| C07 | Malignant neoplasm of parotid gland |
| C08 | Malignant neoplasm of other and unspecified major salivary glands |
| C080 | Malignant neoplasm: Submandibular gland |
| C081 | Malignant neoplasm: Sublingual gland |
| C088 | Malignant neoplasm: Overlapping lesion of major salivary glands |
| C089 | Malignant neoplasm: Major salivary gland, unspecified |
| C09 | Malignant neoplasm of tonsil |
| C090 | Malignant neoplasm: Tonsillar fossa |
| C091 | Malignant neoplasm: Tonsillar pillar (anterior)(posterior) |
| C098 | Malignant neoplasm: Overlapping lesion of tonsil |
| C099 | Malignant neoplasm: Tonsil, unspecified |
| C10 | Malignant neoplasm of oropharynx |
| C100 | Malignant neoplasm: Vallecula |
| C101 | Malignant neoplasm: Anterior surface of epiglottis |
| C102 | Malignant neoplasm: Lateral wall of oropharynx |
| C103 | Malignant neoplasm: Posterior wall of oropharynx |
| C104 | Malignant neoplasm: Branchial cleft |
| C108 | Malignant neoplasm: Overlapping lesion of oropharynx |
| C109 | Malignant neoplasm: Oropharynx, unspecified |
| C11 | Malignant neoplasm of nasopharynx |
| C110 | Malignant neoplasm: Superior wall of nasopharynx |
| C111 | Malignant neoplasm: Posterior wall of nasopharynx |
| C112 | Malignant neoplasm: Lateral wall of nasopharynx |
| C113 | Malignant neoplasm: Anterior wall of nasopharynx |
| C118 | Malignant neoplasm: Overlapping lesion of nasopharynx |
| C119 | Malignant neoplasm: Nasopharynx, unspecified |
| C12 | Malignant neoplasm of piriform sinus |
| C13 | Malignant neoplasm of hypopharynx |
| C130 | Malignant neoplasm: Postcricoid region |
| C131 | Malignant neoplasm: Aryepiglottic fold, hypopharyngeal aspect |
| C132 | Malignant neoplasm: Posterior wall of hypopharynx |
| C138 | Malignant neoplasm: Overlapping lesion of hypopharynx |
| C139 | Malignant neoplasm: Hypopharynx, unspecified |
| C14 | Malignant neoplasm of other and ill-defined sites in the lip, oral cavity and pharynx |
| C140 | Malignant neoplasm: Pharynx, unspecified |
| C142 | Malignant neoplasm: Waldeyer ring |
| C148 | Malignant neoplasm: Overlapping lesion of lip, oral cavity and pharynx |
| C15 | Malignant neoplasm of oesophagus |
| C150 | Malignant neoplasm: Cervical part of oesophagus |
| C151 | Malignant neoplasm: Thoracic part of oesophagus |
| C152 | Malignant neoplasm: Abdominal part of oesophagus |
| C153 | Malignant neoplasm: Upper third of oesophagus |
| C154 | Malignant neoplasm: Middle third of oesophagus |
| C155 | Malignant neoplasm: Lower third of oesophagus |
| C158 | Malignant neoplasm: Overlapping lesion of oesophagus |
| C159 | Malignant neoplasm: Oesophagus, unspecified |
| C16 | Malignant neoplasm of stomach |
| C160 | Malignant neoplasm: Cardia |
| C161 | Malignant neoplasm: Fundus of stomach |
| C162 | Malignant neoplasm: Body of stomach |
| C163 | Malignant neoplasm: Pyloric antrum |
| C164 | Malignant neoplasm: Pylorus |
| C165 | Malignant neoplasm: Lesser curvature of stomach, unspecified |
| C166 | Malignant neoplasm: Greater curvature of stomach, unspecified |
| C168 | Malignant neoplasm: Overlapping lesion of stomach |
| C169 | Malignant neoplasm: Stomach, unspecified |
| C17 | Malignant neoplasm of small intestine |
| C170 | Malignant neoplasm: Duodenum |
| C171 | Malignant neoplasm: Jejunum |
| C172 | Malignant neoplasm: Ileum |
| C173 | Malignant neoplasm: Meckel diverticulum |
| C178 | Malignant neoplasm: Overlapping lesion of small intestine |
| C179 | Malignant neoplasm: Small intestine, unspecified |
| C18 | Malignant neoplasm of colon |
| C180 | Malignant neoplasm: Caecum |
| C181 | Malignant neoplasm: Appendix |
| C182 | Malignant neoplasm: Ascending colon |
| C183 | Malignant neoplasm: Hepatic flexure |
| C184 | Malignant neoplasm: Transverse colon |
| C185 | Malignant neoplasm: Splenic flexure |
| C186 | Malignant neoplasm: Descending colon |
| C187 | Malignant neoplasm: Sigmoid colon |
| C188 | Malignant neoplasm: Overlapping lesion of colon |
| C189 | Malignant neoplasm: Colon, unspecified |
| C19 | Malignant neoplasm of rectosigmoid junction |
| C20 | Malignant neoplasm of rectum |
| C21 | Malignant neoplasm of anus and anal canal |
| C210 | Malignant neoplasm: Anus, unspecified |
| C211 | Malignant neoplasm: Anal canal |
| C212 | Malignant neoplasm: Cloacogenic zone |
| C218 | Malignant neoplasm: Overlapping lesion of rectum, anus and anal canal |
| C22 | Malignant neoplasm of liver and intrahepatic bile ducts |
| C220 | Malignant neoplasm: Liver cell carcinoma |
| C221 | Malignant neoplasm: Intrahepatic bile duct carcinoma |
| C222 | Malignant neoplasm: Hepatoblastoma |
| C223 | Malignant neoplasm: Angiosarcoma of liver |
| C224 | Malignant neoplasm: Other sarcomas of liver |
| C227 | Malignant neoplasm: Other specified carcinomas of liver |
| C229 | Malignant neoplasm: Liver, unspecified |
| C23 | Malignant neoplasm of gallbladder |
| C24 | Malignant neoplasm of other and unspecified parts of biliary tract |
| C240 | Malignant neoplasm: Extrahepatic bile duct |
| C241 | Malignant neoplasm: Ampulla of Vater |
| C248 | Malignant neoplasm: Overlapping lesion of biliary tract |
| C249 | Malignant neoplasm: Biliary tract, unspecified |
| C25 | Malignant neoplasm of pancreas |
| C250 | Malignant neoplasm: Head of pancreas |
| C251 | Malignant neoplasm: Body of pancreas |
| C252 | Malignant neoplasm: Tail of pancreas |
| C253 | Malignant neoplasm: Pancreatic duct |
| C254 | Malignant neoplasm: Endocrine pancreas |
| C257 | Malignant neoplasm: Other parts of pancreas |
| C258 | Malignant neoplasm: Overlapping lesion of pancreas |
| C259 | Malignant neoplasm: Pancreas, unspecified |
| C26 | Malignant neoplasm of other and ill-defined digestive organs |
| C260 | Malignant neoplasm: Intestinal tract, part unspecified |
| C261 | Malignant neoplasm: Spleen |
| C268 | Malignant neoplasm: Overlapping lesion of digestive system |
| C269 | Malignant neoplasm: Ill-defined sites within the digestive system |
| C30 | Malignant neoplasm of nasal cavity and middle ear |
| C300 | Malignant neoplasm: Nasal cavity |
| C301 | Malignant neoplasm: Middle ear |
| C31 | Malignant neoplasm of accessory sinuses |
| C310 | Malignant neoplasm: Maxillary sinus |
| C311 | Malignant neoplasm: Ethmoidal sinus |
| C312 | Malignant neoplasm: Frontal sinus |
| C313 | Malignant neoplasm: Sphenoidal sinus |
| C318 | Malignant neoplasm: Overlapping lesion of accessory sinuses |
| C319 | Malignant neoplasm: Accessory sinus, unspecified |
| C32 | Malignant neoplasm of larynx |
| C320 | Malignant neoplasm: Glottis |
| C321 | Malignant neoplasm: Supraglottis |
| C322 | Malignant neoplasm: Subglottis |
| C323 | Malignant neoplasm: Laryngeal cartilage |
| C328 | Malignant neoplasm: Overlapping lesion of larynx |
| C329 | Malignant neoplasm: Larynx, unspecified |
| C33 | Malignant neoplasm of trachea |
| C34 | Malignant neoplasm of bronchus and lung |
| C340 | Malignant neoplasm: Main bronchus |
| C341 | Malignant neoplasm: Upper lobe, bronchus or lung |
| C342 | Malignant neoplasm: Middle lobe, bronchus or lung |
| C343 | Malignant neoplasm: Lower lobe, bronchus or lung |
| C348 | Malignant neoplasm: Overlapping lesion of bronchus and lung |
| C349 | Malignant neoplasm: Bronchus or lung, unspecified |
| C37 | Malignant neoplasm of thymus |
| C38 | Malignant neoplasm of heart, mediastinum and pleura |
| C380 | Malignant neoplasm: Heart |
| C381 | Malignant neoplasm: Anterior mediastinum |
| C382 | Malignant neoplasm: Posterior mediastinum |
| C383 | Malignant neoplasm: Mediastinum, part unspecified |
| C384 | Malignant neoplasm: Pleura |
| C388 | Malignant neoplasm: Overlapping lesion of heart, mediastinum and pleura |
| C39 | Malignant neoplasm of other and ill-defined sites in the respiratory system and intrathoracic organs |
| C390 | Malignant neoplasm: Upper respiratory tract, part unspecified |
| C398 | Malignant neoplasm: Overlapping lesion of respiratory and intrathoracic organs |
| C399 | Malignant neoplasm: Ill-defined sites within the respiratory system |
| C40 | Malignant neoplasm of bone and articular cartilage of limbs |
| C400 | Malignant neoplasm: Scapula and long bones of upper limb |
| C401 | Malignant neoplasm: Short bones of upper limb |
| C402 | Malignant neoplasm: Long bones of lower limb |
| C403 | Malignant neoplasm: Short bones of lower limb |
| C408 | Malignant neoplasm: Overlapping lesion of bone and articular cartilage of limbs |
| C409 | Malignant neoplasm: Bone and articular cartilage of limb, unspecified |
| C41 | Malignant neoplasm of bone and articular cartilage of other and unspecified sites |
| C410 | Malignant neoplasm: Bones of skull and face |
| C411 | Malignant neoplasm: Mandible |
| C412 | Malignant neoplasm: Vertebral column |
| C413 | Malignant neoplasm: Ribs, sternum and clavicle |
| C414 | Malignant neoplasm: Pelvic bones, sacrum and coccyx |
| C418 | Malignant neoplasm: Overlapping lesion of bone and articular cartilage |
| C419 | Malignant neoplasm: Bone and articular cartilage, unspecified |
| C43 | Malignant melanoma of skin |
| C430 | Malignant neoplasm: Malignant melanoma of lip |
| C431 | Malignant neoplasm: Malignant melanoma of eyelid, including canthus |
| C432 | Malignant neoplasm: Malignant melanoma of ear and external auricular canal |
| C433 | Malignant neoplasm: Malignant melanoma of other and unspecified parts of face |
| C434 | Malignant neoplasm: Malignant melanoma of scalp and neck |
| C435 | Malignant neoplasm: Malignant melanoma of trunk |
| C436 | Malignant neoplasm: Malignant melanoma of upper limb, including shoulder |
| C437 | Malignant neoplasm: Malignant melanoma of lower limb, including hip |
| C438 | Malignant neoplasm: Overlapping malignant melanoma of skin |
| C439 | Malignant neoplasm: Malignant melanoma of skin, unspecified |
| C44 | Other malignant neoplasms of skin |
| C440 | Malignant neoplasm: Skin of lip |
| C441 | Malignant neoplasm: Skin of eyelid, including canthus |
| C442 | Malignant neoplasm: Skin of ear and external auricular canal |
| C443 | Malignant neoplasm: Skin of other and unspecified parts of face |
| C444 | Malignant neoplasm: Skin of scalp and neck |
| C445 | Malignant neoplasm: Skin of trunk |
| C446 | Malignant neoplasm: Skin of upper limb, including shoulder |
| C447 | Malignant neoplasm: Skin of lower limb, including hip |
| C448 | Malignant neoplasm: Overlapping lesion of skin |
| C449 | Malignant neoplasm: Malignant neoplasm of skin, unspecified |
| C45 | Mesothelioma |
| C450 | Mesothelioma of pleura |
| C451 | Mesothelioma of peritoneum |
| C452 | Mesothelioma of pericardium |
| C457 | Mesothelioma of other sites |
| C459 | Mesothelioma, unspecified |
| C46 | Kaposi sarcoma |
| C460 | Kaposi sarcoma of skin |
| C461 | Kaposi sarcoma of soft tissue |
| C462 | Kaposi sarcoma of palate |
| C463 | Kaposi sarcoma of lymph nodes |
| C467 | Kaposi sarcoma of other sites |
| C468 | Kaposi sarcoma of multiple organs |
| C469 | Kaposi sarcoma, unspecified |
| C47 | Malignant neoplasm of peripheral nerves and autonomic nervous system |
| C470 | Malignant neoplasm: Peripheral nerves of head, face and neck |
| C471 | Malignant neoplasm: Peripheral nerves of upper limb, including shoulder |
| C472 | Malignant neoplasm: Peripheral nerves of lower limb, including hip |
| C473 | Malignant neoplasm: Peripheral nerves of thorax |
| C474 | Malignant neoplasm: Peripheral nerves of abdomen |
| C475 | Malignant neoplasm: Peripheral nerves of pelvis |
| C476 | Malignant neoplasm: Peripheral nerves of trunk, unspecified |
| C478 | Malignant neoplasm: Overlapping lesion of peripheral nerves and autonomic nervous system |
| C479 | Malignant neoplasm: Peripheral nerves and autonomic nervous system, unspecified |
| C48 | Malignant neoplasm of retroperitoneum and peritoneum |
| C480 | Malignant neoplasm: Retroperitoneum |
| C481 | Malignant neoplasm: Specified parts of peritoneum |
| C482 | Malignant neoplasm: Peritoneum, unspecified |
| C488 | Malignant neoplasm: Overlapping lesion of retroperitoneum and peritoneum |
| C49 | Malignant neoplasm of other connective and soft tissue |
| C490 | Malignant neoplasm: Connective and soft tissue of head, face and neck |
| C491 | Malignant neoplasm: Connective and soft tissue of upper limb, including shoulder |
| C492 | Malignant neoplasm: Connective and soft tissue of lower limb, including hip |
| C493 | Malignant neoplasm: Connective and soft tissue of thorax |
| C494 | Malignant neoplasm: Connective and soft tissue of abdomen |
| C495 | Malignant neoplasm: Connective and soft tissue of pelvis |
| C496 | Malignant neoplasm: Connective and soft tissue of trunk, unspecified |
| C498 | Malignant neoplasm: Overlapping lesion of connective and soft tissue |
| C499 | Malignant neoplasm: Connective and soft tissue, unspecified |
| C50 | Malignant neoplasm of breast |
| C500 | Malignant neoplasm: Nipple and areola |
| C501 | Malignant neoplasm: Central portion of breast |
| C502 | Malignant neoplasm: Upper-inner quadrant of breast |
| C503 | Malignant neoplasm: Lower-inner quadrant of breast |
| C504 | Malignant neoplasm: Upper-outer quadrant of breast |
| C505 | Malignant neoplasm: Lower-outer quadrant of breast |
| C506 | Malignant neoplasm: Axillary tail of breast |
| C508 | Malignant neoplasm: Overlapping lesion of breast |
| C509 | Malignant neoplasm: Breast, unspecified |
| C51 | Malignant neoplasm of vulva |
| C510 | Malignant neoplasm: Labium majus |
| C511 | Malignant neoplasm: Labium minus |
| C512 | Malignant neoplasm: Clitoris |
| C518 | Malignant neoplasm: Overlapping lesion of vulva |
| C519 | Malignant neoplasm: Vulva, unspecified |
| C52 | Malignant neoplasm of vagina |
| C53 | Malignant neoplasm of cervix uteri |
| C530 | Malignant neoplasm: Endocervix |
| C531 | Malignant neoplasm: Exocervix |
| C538 | Malignant neoplasm: Overlapping lesion of cervix uteri |
| C539 | Malignant neoplasm: Cervix uteri, unspecified |
| C54 | Malignant neoplasm of corpus uteri |
| C540 | Malignant neoplasm: Isthmus uteri |
| C541 | Malignant neoplasm: Endometrium |
| C542 | Malignant neoplasm: Myometrium |
| C543 | Malignant neoplasm: Fundus uteri |
| C548 | Malignant neoplasm: Overlapping lesion of corpus uteri |
| C549 | Malignant neoplasm: Corpus uteri, unspecified |
| C55 | Malignant neoplasm of uterus, part unspecified |
| C56 | Malignant neoplasm of ovary |
| C57 | Malignant neoplasm of other and unspecified female genital organs |
| C570 | Malignant neoplasm: Fallopian tube |
| C571 | Malignant neoplasm: Broad ligament |
| C572 | Malignant neoplasm: Round ligament |
| C573 | Malignant neoplasm: Parametrium |
| C574 | Malignant neoplasm: Uterine adnexa, unspecified |
| C577 | Malignant neoplasm: Other specified female genital organs |
| C578 | Malignant neoplasm: Overlapping lesion of female genital organs |
| C579 | Malignant neoplasm: Female genital organ, unspecified |
| C58 | Malignant neoplasm of placenta |
| C60 | Malignant neoplasm of penis |
| C600 | Malignant neoplasm: Prepuce |
| C601 | Malignant neoplasm: Glans penis |
| C602 | Malignant neoplasm: Body of penis |
| C608 | Malignant neoplasm: Overlapping lesion of penis |
| C609 | Malignant neoplasm: Penis, unspecified |
| C61 | Malignant neoplasm of prostate |
| C62 | Malignant neoplasm of testis |
| C620 | Malignant neoplasm: Undescended testis |
| C621 | Malignant neoplasm: Descended testis |
| C629 | Malignant neoplasm: Testis, unspecified |
| C63 | Malignant neoplasm of other and unspecified male genital organs |
| C630 | Malignant neoplasm: Epididymis |
| C631 | Malignant neoplasm: Spermatic cord |
| C632 | Malignant neoplasm: Scrotum |
| C637 | Malignant neoplasm: Other specified male genital organs |
| C638 | Malignant neoplasm: Overlapping lesion of male genital organs |
| C639 | Malignant neoplasm: Male genital organ, unspecified |
| C64 | Malignant neoplasm of kidney, except renal pelvis |
| C65 | Malignant neoplasm of renal pelvis |
| C66 | Malignant neoplasm of ureter |
| C67 | Malignant neoplasm of bladder |
| C670 | Malignant neoplasm: Trigone of bladder |
| C671 | Malignant neoplasm: Dome of bladder |
| C672 | Malignant neoplasm: Lateral wall of bladder |
| C673 | Malignant neoplasm: Anterior wall of bladder |
| C674 | Malignant neoplasm: Posterior wall of bladder |
| C675 | Malignant neoplasm: Bladder neck |
| C676 | Malignant neoplasm: Ureteric orifice |
| C677 | Malignant neoplasm: Urachus |
| C678 | Malignant neoplasm: Overlapping lesion of bladder |
| C679 | Malignant neoplasm: Bladder, unspecified |
| C68 | Malignant neoplasm of other and unspecified urinary organs |
| C680 | Malignant neoplasm: Urethra |
| C681 | Malignant neoplasm: Paraurethral gland |
| C688 | Malignant neoplasm: Overlapping lesion of urinary organs |
| C689 | Malignant neoplasm: Urinary organ, unspecified |
| C69 | Malignant neoplasm of eye and adnexa |
| C690 | Malignant neoplasm: Conjunctiva |
| C691 | Malignant neoplasm: Cornea |
| C692 | Malignant neoplasm: Retina |
| C693 | Malignant neoplasm: Choroid |
| C694 | Malignant neoplasm: Ciliary body |
| C695 | Malignant neoplasm: Lacrimal gland and duct |
| C696 | Malignant neoplasm: Orbit |
| C698 | Malignant neoplasm: Overlapping lesion of eye and adnexa |
| C699 | Malignant neoplasm: Eye, unspecified |
| C70 | Malignant neoplasm of meninges |
| C700 | Malignant neoplasm: Cerebral meninges |
| C701 | Malignant neoplasm: Spinal meninges |
| C709 | Malignant neoplasm: Meninges, unspecified |
| C71 | Malignant neoplasm of brain |
| C710 | Malignant neoplasm: Cerebrum, except lobes and ventricles |
| C711 | Malignant neoplasm: Frontal lobe |
| C712 | Malignant neoplasm: Temporal lobe |
| C713 | Malignant neoplasm: Parietal lobe |
| C714 | Malignant neoplasm: Occipital lobe |
| C715 | Malignant neoplasm: Cerebral ventricle |
| C716 | Malignant neoplasm: Cerebellum |
| C717 | Malignant neoplasm: Brain stem |
| C718 | Malignant neoplasm: Overlapping lesion of brain |
| C719 | Malignant neoplasm: Brain, unspecified |
| C72 | Malignant neoplasm of spinal cord, cranial nerves and other parts of central nervous system |
| C720 | Malignant neoplasm: Spinal cord |
| C721 | Malignant neoplasm: Cauda equina |
| C722 | Malignant neoplasm: Olfactory nerve |
| C723 | Malignant neoplasm: Optic nerve |
| C724 | Malignant neoplasm: Acoustic nerve |
| C725 | Malignant neoplasm: Other and unspecified cranial nerves |
| C728 | Malignant neoplasm: Overlapping lesion of brain and other parts of central nervous system |
| C729 | Malignant neoplasm: Central nervous system, unspecified |
| C73 | Malignant neoplasm of thyroid gland |
| C74 | Malignant neoplasm of adrenal gland |
| C740 | Malignant neoplasm: Cortex of adrenal gland |
| C741 | Malignant neoplasm: Medulla of adrenal gland |
| C749 | Malignant neoplasm: Adrenal gland, unspecified |
| C75 | Malignant neoplasm of other endocrine glands and related structures |
| C750 | Malignant neoplasm: Parathyroid gland |
| C751 | Malignant neoplasm: Pituitary gland |
| C752 | Malignant neoplasm: Craniopharyngeal duct |
| C753 | Malignant neoplasm: Pineal gland |
| C754 | Malignant neoplasm: Carotid body |
| C755 | Malignant neoplasm: Aortic body and other paraganglia |
| C758 | Malignant neoplasm: Pluriglandular involvement, unspecified |
| C759 | Malignant neoplasm: Endocrine gland, unspecified |
| C76 | Malignant neoplasm of other and ill-defined sites |
| C760 | Malignant neoplasm of other and ill-defined sites: Head, face and neck |
| C761 | Malignant neoplasm of other and ill-defined sites: Thorax |
| C762 | Malignant neoplasm of other and ill-defined sites: Abdomen |
| C763 | Malignant neoplasm of other and ill-defined sites: Pelvis |
| C764 | Malignant neoplasm of other and ill-defined sites: Upper limb |
| C765 | Malignant neoplasm of other and ill-defined sites: Lower limb |
| C767 | Malignant neoplasm of other and ill-defined sites: Other ill-defined sites |
| C768 | Malignant neoplasm of other and ill-defined sites: Overlapping lesion of other and ill-defined sites |
| C77 | Secondary and unspecified malignant neoplasm of lymph nodes |
| C770 | Secondary and unspecified malignant neoplasm: Lymph nodes of head, face and neck |
| C771 | Secondary and unspecified malignant neoplasm: Intrathoracic lymph nodes |
| C772 | Secondary and unspecified malignant neoplasm: Intra-abdominal lymph nodes |
| C773 | Secondary and unspecified malignant neoplasm: Axillary and upper limb lymph nodes |
| C774 | Secondary and unspecified malignant neoplasm: Inguinal and lower limb lymph nodes |
| C775 | Secondary and unspecified malignant neoplasm: Intrapelvic lymph nodes |
| C778 | Secondary and unspecified malignant neoplasm: Lymph nodes of multiple regions |
| C779 | Secondary and unspecified malignant neoplasm: Lymph node, unspecified |
| C78 | Secondary malignant neoplasm of respiratory and digestive organs |
| C780 | Secondary malignant neoplasm of lung |
| C781 | Secondary malignant neoplasm of mediastinum |
| C782 | Secondary malignant neoplasm of pleura |
| C783 | Secondary malignant neoplasm of other and unspecified respiratory organs |
| C784 | Secondary malignant neoplasm of small intestine |
| C785 | Secondary malignant neoplasm of large intestine and rectum |
| C786 | Secondary malignant neoplasm of retroperitoneum and peritoneum |
| C787 | Secondary malignant neoplasm of liver and intrahepatic bile duct |
| C788 | Secondary malignant neoplasm of other and unspecified digestive organs |
| C79 | Secondary malignant neoplasm of other and unspecified sites |
| C790 | Secondary malignant neoplasm of kidney and renal pelvis |
| C791 | Secondary malignant neoplasm of bladder and other and unspecified urinary organs |
| C792 | Secondary malignant neoplasm of skin |
| C793 | Secondary malignant neoplasm of brain and cerebral meninges |
| C794 | Secondary malignant neoplasm of other and unspecified parts of nervous system |
| C795 | Secondary malignant neoplasm of bone and bone marrow |
| C796 | Secondary malignant neoplasm of ovary |
| C797 | Secondary malignant neoplasm of adrenal gland |
| C798 | Secondary malignant neoplasm of other specified sites |
| C799 | Secondary malignant neoplasm, unspecified site |
| C80 | Malignant neoplasm without specification of site |
| C800 | Malignant neoplasm, primary site unknown, so stated |
| C809 | Malignant neoplasm, primary site unspecified |
| C81 | Hodgkin lymphoma |
| C810 | Nodular lymphocyte predominant Hodgkin lymphoma |
| C811 | Nodular sclerosis (classical) Hodgkin lymphoma |
| C812 | Mixed cellularity (classical) Hodgkin lymphoma |
| C813 | Lymphocyte depleted (classical) Hodgkin lymphoma |
| C814 | Lymphocyte-rich (classical) Hodgkin lymphoma |
| C817 | Other (classical) Hodgkin lymphoma |
| C819 | Hodgkin lymphoma, unspecified |
| C82 | Follicular lymphoma |
| C820 | Follicular lymphoma grade I |
| C821 | Follicular lymphoma grade II |
| C822 | Follicular lymphoma grade III, unspecified |
| C823 | Follicular lymphoma grade IIIa |
| C824 | Follicular lymphoma grade IIIb |
| C825 | Diffuse follicle centre lymphoma |
| C826 | Cutaneous follicle centre lymphoma |
| C827 | Other types of follicular lymphoma |
| C829 | Follicular lymphoma, unspecified |
| C83 | Non-follicular lymphoma |
| C830 | Small cell B-cell lymphoma |
| C831 | Mantle cell lymphoma |
| C833 | Diffuse large B-cell lymphoma |
| C835 | Lymphoblastic (diffuse) lymphoma |
| C837 | Burkitt lymphoma |
| C838 | Other non-follicular lymphoma |
| C839 | Non-follicular (diffuse) lymphoma, unspecified |
| C84 | Mature T/NK-cell lymphomas |
| C840 | Mycosis fungoides |
| C841 | SÃ©zary disease |
| C844 | Peripheral T-cell lymphoma, not elsewhere classified |
| C845 | Other mature T/NK-cell lymphomas |
| C846 | Anaplastic large cell lymphoma, ALK-positive |
| C847 | Anaplastic large cell lymphoma, ALK-negative |
| C848 | Cutaneous T-cell lymphoma, unspecified |
| C849 | Mature T/NK-cell lymphoma, unspecified |
| C85 | Other and unspecified types of non-Hodgkin lymphoma |
| C851 | B-cell lymphoma, unspecified |
| C852 | Mediastinal (thymic) large B-cell lymphoma |
| C857 | Other specified types of non-Hodgkin lymphoma |
| C859 | Non-Hodgkin lymphoma, unspecified |
| C86 | Other specified types of T/NK-cell lymphoma |
| C860 | Extranodal NK/T-cell lymphoma, nasal type |
| C861 | Hepatosplenic T-cell lymphoma |
| C862 | Enteropathy-type (intestinal) T-cell lymphoma |
| C863 | Subcutaneous panniculitis-like T-cell lymphoma |
| C864 | Blastic NK-cell lymphoma |
| C865 | Angioimmunoblastic T-cell lymphoma |
| C866 | Primary cutaneous CD30-positive T-cell proliferations |
| C88 | Malignant immunoproliferative diseases |
| C880 | WaldenstrÃ¶m macroglobulinaemia |
| C882 | Other heavy chain disease |
| C883 | Immunoproliferative small intestinal disease |
| C884 | Extranodal marginal zone B-cell lymphoma of mucosa-associated lymphoid tissue [MALT-lymphoma] |
| C887 | Other malignant immunoproliferative diseases |
| C889 | Malignant immunoproliferative disease, unspecified |
| C90 | Multiple myeloma and malignant plasma cell neoplasms |
| C900 | Multiple myeloma |
| C901 | Plasma cell leukaemia |
| C902 | Extramedullary plasmacytoma |
| C903 | Solitary plasmacytoma |
| C91 | Lymphoid leukaemia |
| C910 | Acute lymphoblastic leukaemia [ALL] |
| C911 | Chronic lymphocytic leukaemia of B-cell type |
| C913 | Prolymphocytic leukaemia of B-cell type |
| C914 | Hairy-cell leukaemia |
| C915 | Adult T-cell lymphoma/leukaemia [HTLV-1-associated] |
| C916 | Prolymphocytic leukaemia of T-cell type |
| C917 | Other lymphoid leukaemia |
| C918 | Mature B-cell leukaemia Burkitt-type |
| C919 | Lymphoid leukaemia, unspecified |
| C92 | Myeloid leukaemia |
| C920 | Acute myeloblastic leukaemia [AML] |
| C921 | Chronic myeloid leukaemia [CML], BCR/ABL-positive |
| C922 | Atypical chronic myeloid leukaemia, BCR/ABL-negative |
| C923 | Myeloid sarcoma |
| C924 | Acute promyelocytic leukaemia [PML] |
| C925 | Acute myelomonocytic leukaemia |
| C926 | Acute myeloid leukaemia with 11q23-abnormality |
| C927 | Other myeloid leukaemia |
| C928 | Acute myeloid leukaemia with multilineage dysplasia |
| C929 | Myeloid leukaemia, unspecified |
| C93 | Monocytic leukaemia |
| C930 | Acute monoblastic/monocytic leukaemia |
| C931 | Chronic myelomonocytic leukaemia |
| C933 | Juvenile myelomonocytic leukaemia |
| C937 | Other monocytic leukaemia |
| C939 | Monocytic leukaemia, unspecified |
| C94 | Other leukaemias of specified cell type |
| C940 | Acute erythroid leukaemia |
| C942 | Acute megakaryoblastic leukaemia |
| C943 | Mast cell leukaemia |
| C944 | Acute panmyelosis with myelofibrosis |
| C946 | Myelodysplastic and myeloproliferative disease, not elsewhere classified |
| C947 | Other specified leukaemias |
| C95 | Leukaemia of unspecified cell type |
| C950 | Acute leukaemia of unspecified cell type |
| C951 | Chronic leukaemia of unspecified cell type |
| C957 | Other leukaemia of unspecified cell type |
| C959 | Leukaemia, unspecified |
| C96 | Other and unspecified malignant neoplasms of lymphoid, haematopoietic and related tissue |
| C960 | Multifocal and multisystemic (disseminated) Langerhans-cell histiocytosis [Letterer-Siwe disease] |
| C962 | Malignant mast cell tumour |
| C964 | Sarcoma of dendritic cells (accessory cells) |
| C965 | Multifocal and unisystemic Langerhans-cell histiocytosis |
| C966 | Unifocal Langerhans-cell histiocytosis |
| C967 | Other specified malignant neoplasms of lymphoid, haematopoietic and related tissue |
| C968 | Histiocytic sarcoma |
| C969 | Malignant neoplasm of lymphoid, haematopoietic and related tissue, unspecified |
| C97 | Malignant neoplasms of independent (primary) multiple sites |

| **Immunosuppression** | |
| --- | --- |
| D80 | Immunodeficiency with predominantly antibody defects |
| D800 | Hereditary hypogammaglobulinaemia |
| D801 | Nonfamilial hypogammaglobulinaemia |
| D802 | Selective deficiency of immunoglobulin A [IgA] |
| D803 | Selective deficiency of immunoglobulin G [IgG] subclasses |
| D804 | Selective deficiency of immunoglobulin M [IgM] |
| D805 | Immunodeficiency with increased immunoglobulin M [IgM] |
| D806 | Antibody deficiency with near-normal immunoglobulins or with hyperimmunoglobulinaemia |
| D807 | Transient hypogammaglobulinaemia of infancy |
| D808 | Other immunodeficiencies with predominantly antibody defects |
| D809 | Immunodeficiency with predominantly antibody defects, unspecified |
| D81 | Combined immunodeficiencies |
| D810 | Severe combined immunodeficiency [SCID] with reticular dysgenesis |
| D811 | Severe combined immunodeficiency [SCID] with low T- and B-cell numbers |
| D812 | Severe combined immunodeficiency [SCID] with low or normal B-cell numbers |
| D813 | Adenosine deaminase [ADA] deficiency |
| D814 | Nezelof syndrome |
| D815 | Purine nucleoside phosphorylase [PNP] deficiency |
| D816 | Major histocompatibility complex class I deficiency |
| D817 | Major histocompatibility complex class II deficiency |
| D818 | Other combined immunodeficiencies |
| D819 | Combined immunodeficiency, unspecified |
| D82 | Immunodeficiency associated with other major defects |
| D820 | Wiskott-Aldrich syndrome |
| D821 | Di George syndrome |
| D822 | Immunodeficiency with short-limbed stature |
| D823 | Immunodeficiency following hereditary defective response to Epstein-Barr virus |
| D824 | Hyperimmunoglobulin E [IgE] syndrome |
| D828 | Immunodeficiency associated with other specified major defects |
| D829 | Immunodeficiency associated with major defect, unspecified |
| D83 | Common variable immunodeficiency |
| D830 | Common variable immunodeficiency with predominant abnormalities of B-cell numbers and function |
| D831 | Common variable immunodeficiency with predominant immunoregulatory T-cell disorders |
| D832 | Common variable immunodeficiency with autoantibodies to B- or T-cells |
| D838 | Other common variable immunodeficiencies |
| D839 | Common variable immunodeficiency, unspecified |
| D84 | Other immunodeficiencies |
| D840 | Lymphocyte function antigen-1 [LFA-1] defect |
| D841 | Defects in the complement system |
| D848 | Other specified immunodeficiencies |
| D849 | Immunodeficiency, unspecified |
| D86 | Sarcoidosis |
| D860 | Sarcoidosis of lung |
| D861 | Sarcoidosis of lymph nodes |
| D862 | Sarcoidosis of lung with sarcoidosis of lymph nodes |
| D863 | Sarcoidosis of skin |
| D868 | Sarcoidosis of other and combined sites |
| D869 | Sarcoidosis, unspecified |
| D89 | Other disorders involving the immune mechanism, not elsewhere classified |
| D890 | Polyclonal hypergammaglobulinaemia |
| D891 | Cryoglobulinaemia |
| D892 | Hypergammaglobulinaemia, unspecified |
| D893 | Immune reconstitution syndrome |
| D898 | Other specified disorders involving the immune mechanism, not elsewhere classified |
| D899 | Disorder involving the immune mechanism, unspecified |

# Charlson comorbidity index (CCI)

As listed in Table 1 of

Quan, Hude, Vijaya Sundararajan, Patricia Halfon, Andrew Fong, Bernard Burnand, Jean-Christophe Luthi, L. Duncan Saunders, Cynthia A. Beck, Thomas E. Feasby, and William A. Ghali. 2005. “Coding Algorithms for Defining Comorbidities in ICD-9-CM and ICD-10 Administrative Data.” *Medical Care* 43 (11): 1130–39.
